# Prestin-Dependence of Outer Hair Cell Survival and Partial Rescue of Outer Hair Cell Loss in *Prestin* ^V499G/Y501H^ Knockin Mice

**DOI:** 10.1371/journal.pone.0145428

**Published:** 2015-12-18

**Authors:** Mary Ann Cheatham, Roxanne M. Edge, Kazuaki Homma, Emily L. Leserman, Peter Dallos, Jing Zheng

**Affiliations:** 1 The Knowles Hearing Center, Roxelyn and Richard Pepper Department of Communication Sciences and Disorders, Northwestern University, Evanston, IL, 60208, United States of America; 2 The Knowles Hearing Center, Department of Otolaryngology-Head and Neck Surgery, Feinberg School of Medicine, Northwestern University, Chicago, IL, 60611, United States of America; 3 Department of Neurobiology, Northwestern University, Evanston, IL, 60208, United States of America; Harvard University, UNITED STATES

## Abstract

A knockin (KI) mouse expressing mutated *prestin* ^V499G/Y501H^ (499 *prestin*) was created to study cochlear amplification. Recordings from isolated outer hair cells (OHC) in this mutant showed vastly reduced electromotility and, as a consequence, reduced hearing sensitivity. Although 499 *prestin* OHCs were normal in stiffness and longer than OHCs lacking prestin, accelerated OHC death was unexpectedly observed relative to that documented in *prestin* knockout (KO) mice. These observations imply an additional role of prestin in OHC maintenance besides its known requirement for mammalian cochlear amplification. In order to gain mechanistic insights into prestin-associated OHC loss, we implemented several interventions to improve survival. First, 499 *prestin* KI’s were backcrossed to *Bak* KO mice, which lack the mitochondrial pro-apoptotic gene *Bak*. Because oxidative stress is implicated in OHC death, another group of 499 *prestin* KI mice was fed the antioxidant diet, Protandim. 499 KI mice were also backcrossed onto the FVB murine strain, which retains excellent high-frequency hearing well into adulthood, to reduce the compounding effect of age-related hearing loss associated with the original 499 *prestin* KIs. Finally, a compound heterozygous (chet) mouse expressing one copy of 499 *prestin* and one copy of KO *prestin* was also created to reduce quantities of 499 prestin protein. Results show reduction in OHC death in chets, and in 499 *prestin* KIs on the FVB background, but only a slight improvement in OHC survival for mice receiving Protandim. We also report that improved OHC survival in 499 *prestin* KIs had little effect on hearing phenotype, reaffirming the original contention about the essential role of prestin’s motor function in cochlear amplification.

## Introduction

Prestin, the molecular motor essential for feedback amplification in the cochlea [1,5] is exclusively expressed in outer hair cells (OHCs) and is required for electromechanical (reverse) transduction. In order to understand prestin’s role in OHC electromotility, a mouse model was created in which the *prestin* gene was targeted for deletion. Cochlear morphology in the *prestin* null was normal, except for a truncation in OHC length and premature loss of OHCs in the basal 25% of the cochlea [1,3]. OHCs lacking prestin had no measureable motility, threshold shifts were ~50 dB [1] and tuning functions lacked sharp tip segments [6]. Although these results indicate that prestin is required for OHC electromotility, it is difficult to determine on their bases the degree to which prestin contributes to cochlear amplification due to structural and mechanical changes in the KO organ of Corti. OHCs in *prestin* KO mice are only 60% of WT in length [7] and their stiffness is reduced [2]. These changes in OHC properties influence the load seen by the amplifier with the result that the complex feedback loop including the basilar membrane, OHC and tectorial membrane is altered. These changes in physical/anatomical properties could well result in a loss of gain independent of whether prestin was responsible for amplification [8].

In order to circumvent these difficulties, a *prestin* knockin (KI) mouse was developed by altering amino acids, V499G and Y501H, which reside near the presumed junction between prestin’s last transmembrane domain and its intracellular C terminus [1]. The substitutions were made because of previous work showing that 499 prestin targeted the membrane but displayed significantly diminished functional characteristics, i.e., nonlinear capacitance (NLC) [9]. It was also demonstrated that mutation of amino acid 499 was solely responsible for the change in phenotype and that 499 prestin is a slow motor [10], making it nonfunctional in mice. Although sensitivity decreased and frequency selectivity was reduced in 499 *prestin* KI mice, forward transduction and fast adaptation were WT-like, implying that a putative hair-bundle amplifier should still be operational. Hence, these results are consistent with the idea that prestin is required for cochlear amplification (Dallos et al. 2008). In this report, we provide additional information including the unexpected finding that 499 *prestin* KIs suffer aggressive OHC death even though the OHCs retain their stiffness and the cells contain a full complement of prestin, albeit modified.

Because the phenotype of mice without OHCs [11–13] is similar to that for OHCs lacking prestin, it is necessary to develop interventions that enhance hair-cell preservation in order to improve the utility of *prestin* mouse models. This is especially important in 499 *prestin* KI mice since they retain a normal anatomical/physical structure. Consequently, we designed a series of experiments to evaluate various interventions that promised to extend cell life [14]. In the first intervention, 499 *prestin* KI mice were created with a deletion of the mitochondrial pro-apoptotic gene *Bak*, in order to delay entry into an apoptotic cell-death pathway. In normal mice, a mitochondrially-targeted catalase (MCAT) suppresses *Bak* expression in the cochlea, thereby reducing DNA damage associated with oxidative stress, and delaying the onset of age-related hearing loss (AHL). In fact, overexpression of catalase has been shown to reduce AHL, consistent with the idea that mitochondria-derived reactive oxygen species (ROS) play a role [15].

Someya et al. (2009) also reported that mitochondrial antioxidant supplementation reduces pro-apoptotic *Bak* expression and improves hair-cell survival, thereby delaying the onset of AHL. This information, as well as the growing implication of oxidative stress in hair-cell death and neural degeneration [16–19], prompted us to include a mouse model that had been raised on an anti-oxidant diet. Protandim, a new antioxidant approach in chemoprevention, increases the expression of superoxide dismutase [20] and catalase activities, thereby decreasing superoxide generation and lipid peroxidation [4]. Since it is known that oxidative stress increases with age in C57BL/6J mice, supplementing the mouse diet with Protandim might reduce oxidative damage and OHC loss in 499 prestin KI mice.

A third intervention involved back crossing onto the FVB murine strain, which exhibits good hearing well into adulthood in contrast to the strains originally used to develop the 499 *prestin* KI mouse (129S6/C57BL6). Finally, a compound heterozygote (chet) with one copy of 499 *prestin* and one copy of a mutated allele producing no prestin protein, i.e., the knockout/null allele, was also developed. If there were no upregulation of mutated 499 prestin protein expression, then chets would be expected to generate about half as much 499 prestin protein when compared to OHCs in mice that are homozygous for this mutated allele. If OHC death in 499 *prestin* KIs is intrinsic rather than being triggered by extrinsic factors, then perhaps a reduction in 499 prestin protein expression may increase OHC survival.

## Materials and Methods

### Animals

All work was approved by Northwestern University’s Institutional Animal Care and Use Committee (IACUC) and by the National Institutes of Health. Data were collected from mice with mutations in the *prestin* gene (V499G/Y501H, referred to as 499 *prestin* KI) that were originally developed on a mixed 129S6/C57BL6 (129/B6) genetic background. Although the initial report on this mouse model [2] provided results on generations F3/4, most of the data presented here were obtained from later generations maintained as hetero- or homo-typic breeding. One group was fed an antioxidant diet, Protandim (n = 6). In this case, 499 *prestin* KI mice received a modified AIN-93G Growth Purified Rodent Diet supplemented with 1200 mg/kg Protandim, while control animals received an unmodified AIN-93G diet. Both formulations were provided by Diets, Inc. (Bethlehem PA) since this company supplied the Protandim diet to Liu and colleagues [4] for their work on chemoprevention. 499 KI breeder pairs were fed the Protandim diet well before pups were born in order to assure that the antioxidant-rich diet was available in utero. Secondly, *Bak* knockout (KO) mice (Jackson Laboratories #004183) on a C57BL6J background and lacking the mitochondrial pro-apoptotic gene *Bak* (n = 5) were crossed with 499 KI mice to obtain KIs that were also lacking this pro-apoptotic gene. 499 KI mice were also backcrossed onto the FVB strain (albino), which lacks known AHL genes (n = 6). In order to obtain a 499 *prestin* KI that was at least 95% of the recipient FVB strain, data were collected on 499/FVB KI mice at generation N8. It should be understood, however, that speed congenics was not undertaken. Finally, we created chets by crossing either C1 *prestin* KI [21,22] or 499 *prestin* KI mice with *prestin* KOs [1] to examine the degree to which mutated *prestin* might be up regulated.

### Tissue processing and cochleogram construction

Animals were euthanized at ~6 weeks of age (P42) and cardiac perfused first with heparinized saline and then with 4% paraformaldehyde in 0.01 M phosphate-buffered saline (PBS). Cochleae were post fixed for 2–4 hours at room temperature and then rinsed in 0.01M PBS (4 times, 15 min each) followed by a second rinse with 50 mM Tris-buffered saline (TBS, 4 times for 5 min each). After TBS removal, the specimens were placed in 0.3% Triton X-100 for one hour to open holes in the plasma membrane since the prestin antibody is designed to recognize the intracellular C terminal domain.

After rinsing in 0.01% ovalbumin, specimens were placed in blocking solution (10% ovalbumin, 5% BSA in TBS) for at least 1 hour. The primary prestin antibody (1:1000) was then perfused through each cochlea, which was then placed in a refrigerator over night. The next day, cochleae were perfused with horseradish peroxidase (HRP) tagged, anti-rabbit IgG (1:2000). After rinsing, cochleae were placed in diaminobenzidine (DAB) to produce a brown reaction product. Finally, cochleae were rinsed before plastic embedding (Epon-Araldite). The embedding procedure involved dehydration in acetone plus an infiltration stage prior to curing overnight at 60°C.

Embedded cochleae were cut into segments and trimmed of excess plastic before mounting individual segments onto glass slides for examination. Using MicroSuite FIVE (Olympus Soft Imaging Solutions) images were taken of the entire cochlea from apex to base and a composite created by stitching individual images into a montage using “DoubleTake” (Echo One). Measurements were then made to determine if basilar membrane (BM) length was within one standard deviation of the mean confirming that the stitching process was performed adequately (average basilar membrane length for 129/C57BL6 mice is 5.86 +/- 0.08 mm; for FVB 5.39 +/- 0.13 mm). The BM was then divided into 7% sections beginning at the base, which leaves a 2% remnant at the apical end. This procedure allows us to compare our estimates of OHC death with those of Wu et al. [3] on the original *prestin* KO. Within each 7% section, counts were made to determine the number of hair cells present/absent. These values were then used to create cochleograms where percent cell death was plotted as a function of percent distance from the apex in order to document the location and degree of OHC loss [23]. Because previous results [3], as well as our own unpublished data, indicate no significant difference in OHC death for individual rows, the cochleograms provide a single datum for all 3 rows of OHCs. Means and standard deviations are plotted for all experimental conditions. A Student’s t-test was performed to determine whether a given treatment ameliorated OHC death in 499 *prestin* KI mice. OHC lengths were measured by re-embedding tissue at a position that was either ~2.1 (~13 kHz place), or 0.5 (~6 kHz place) mm from the helicotrema [24] at both P21 and P42. 5-micron radial sections were then used to measure cell length from the top of the cuticular plate to the bottom of the OHC nucleus.

### Physiological assessments

Distortion product otoacoustic emissions (DPOAE) were collected in mice less than 8 weeks of age to assay changes in sensitivity. These results were obtained as input-output functions for various f2 frequencies where f2/f1 was 1.2. In order to maximize the DPOAE at 2f1-f2, the level of f1 was 10 dB greater than that for f2. Data were also collected as DPgrams where the parameter was f2 frequency. In this case, the two primaries were presented at equal levels (L1 = L2 = 70 dB). Auditory brainstem responses (ABR) were also collected and thresholds determined by noting the signal level where the ABR waveform disappeared into the noise floor. All physiological measurements were obtained in mice anesthetized with ketamine/xylazine. Additional details are provided in a previous publication [25].

## Results

### 499 prestin KI mice suffer more aggressive OHC loss than prestin KO mice

**Fig 1:**
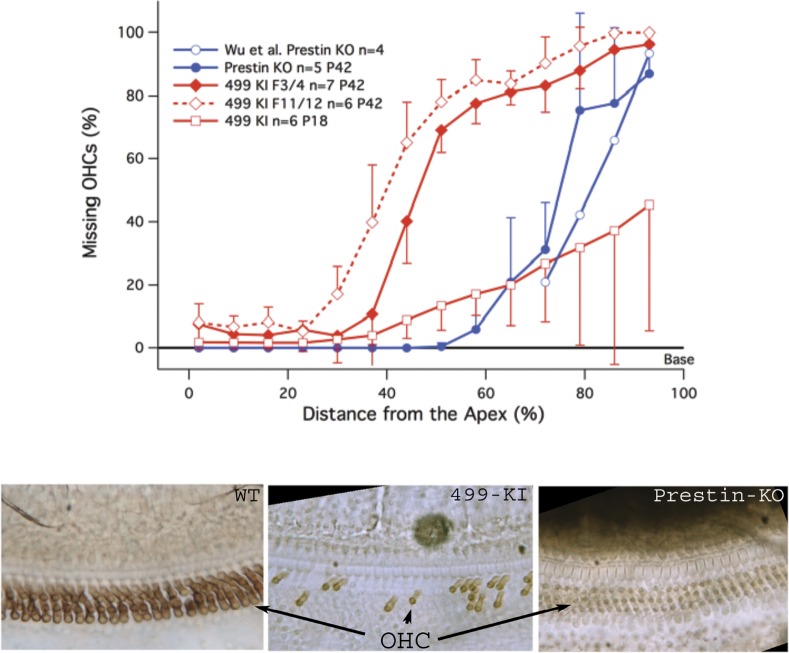
Hair cell loss in *prestin* KO and 499 *prestin* KI mice. The top panel summarizes cochleograms constructed for the original *prestin* KO mouse (blue) and for the 499 *prestin* KI (red). The results from Wu et al. (3) are replotted (blue open circles) along with our confirmation of OHC death in mice lacking prestin protein (blue solid circles). Since these results were obtained at P42, the 499 *prestin* KI values are also collected at 6 weeks of age. The red solid line shows OHCs missing for generations F3/4, while the dashed line shows the results for generations F11/12. (The F3/4 generation was that used in the original study by Dallos and colleagues). Because of the aggressive cell death in mice expressing 499 *prestin*, cochleograms were also constructed for mice at P18 (red solid line with open squares). Means and standard deviations are shown. The images in the lower panel were obtained from representative WT, 499 *prestin* KI and prestin KO mice all at P42 and all obtained at the 50% location.

It is known that *prestin* KO mice exhibit OHC loss [1] but in mice less than 8 weeks of age the loss is restricted to the basal 25% of the cochlea. More importantly, the loss of OHCs occurs after weaning and after the mice show threshold shift due to lack of prestin [3]. These latter results are replotted in Fig 1 with open blue circles at P42. Our measurements (solid blue circles) confirm the original findings. Also provided in red are the cochleograms for early (F3/4, solid diamonds) and late generation (F11/12, open diamonds) 499 *prestin* KI mice also at P42. The boundary between OHCs present and absent moves more apically in late generation animals. Because of the accelerated cell death in 499 *prestin* KIs, we also collected data at P18 (F11/12) in order to determine if cell death might contribute to the change in hearing phenotype. Even in these younger animals there are missing OHCs over a wide region of the cochlea, implying that 499 KIs suffer OHC death before cochlear development is complete. We also observed no missing OHCs at P12 when mice have little ability to process even high-level signals. Although “motor particles” appear in the lateral OHC membrane as early as P2 [26], electromotility is not fully functional until ~P14 [27–29]. This information implies that the onset of motor action may impact OHC survival in mice expressing mutant prestin protein.

To demonstrate the stark comparison between the numbers of surviving OHCs in *prestin* KO versus 499 *prestin* KI mice, the lower panel of Fig 1 provides whole-mount images of the organ of Corti focusing down from the scala media side at a location that is halfway between apex and base. Both WT and prestin KO samples look normal at P42 in contrast to tissue in 499 KIs where only a few OHCs stain brown when using a prestin antibody with a peroxidase tag.

In contrast to IHC loss, which is less than 5% in all mutants examined so far, the average percent missing OHCs in 499 *prestin* KI late generation (F11/12) mice is 54.32 ± 4.86% at P42. Early generation (F3/4) 499 *prestin* KIs have 46.79 ± 4.93% missing OHCs per cochlea. Even at ~P18, the 499 *prestin* KIs are missing 20.9% ± 11.8% of their OHCs. Although more variable, this latter datum is similar to that observed in early generation *prestin* KO mice (F3/4) at P42 where 20.54 ± 2.10% of the OHCs in a given cochlea are absent. Because 499 *prestin* KI mice unexpectedly showed accelerated OHC death, several efforts were made to ameliorate this loss using 499 KI late generation mice as controls.

### Removal of the pro-apoptotic gene, Bak

**Fig 2:**
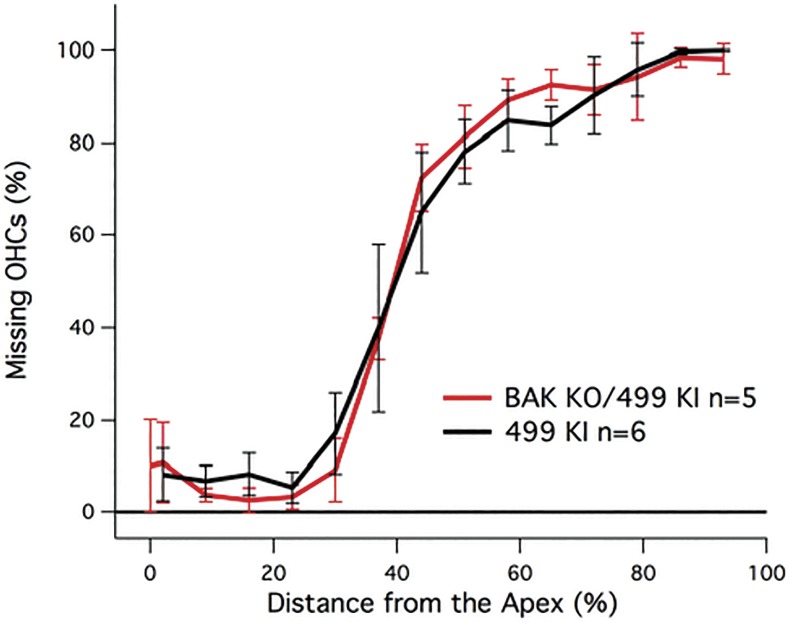
Cochleograms for Bak KO/499 *prestin* KI versus 499 *prestin* KI (F11/12) mice. Percent missing OHCs are plotted as a function of distance from the apex. Data for the 499 *prestin* KIs are plotted in black.

Because apoptosis is implicated in OHC death in many cases [14,30], 499 *prestin* KIs were crossed with *Bak* KO mice on a C57BL6 background, which lack the mitochondrial pro-apoptotic gene *Bak*. At P42, the average percent missing OHCs in the *Bak*-KO/499 *prestin* KI mice was 54.3 ± 2.7% per cochlea. Cochleograms for the BAK KO/499 *prestin* KI mice (n = 5) and their controls are plotted in Fig 2 and show no improvement in OHC death using this approach. We also used minocycline, a tetracycline derived antibiotic that prevents activation of caspases during apoptosis, a cell death pathway implicated in OHC loss due to aminoglycosides [31,32]. No improvement in OHC survival was observed.

### Anti-oxidant supplementation using the Protandim diet

**Fig 3:**
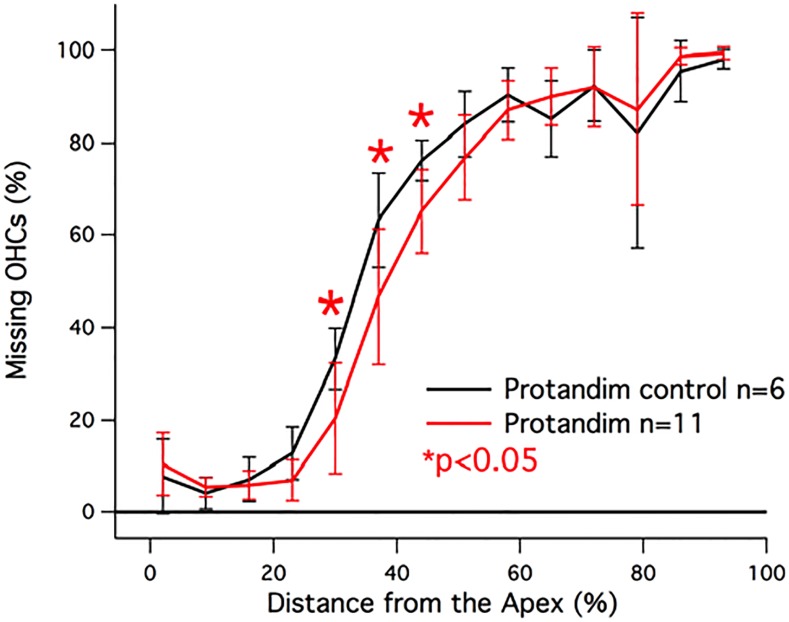
Protandim versus the control diet. Mice receiving Protandim are plotted in red and the mice fed the control diet in black. Improvement in OHC survival was statistically significant (p<0.05) for sections 30, 37, and 44% distance from the apex, as demarcated by the asterisks.

Since several experiments suggested that reducing ROS increases OHC survival, we used external anti-oxidants directly such as d-methionine [33,34], and a vitamin-enriched diet [35] in an attempt to reduce OHC loss in 499-KI mice. An improvement in OHC survival was not observed for these treatments when animals were sacrificed at P42. Subsequently, 499 *prestin* KI mice were fed an antioxidant diet (Protandim, n = 11), which is reported to increase internal catalase activity as well as the expression levels of superoxide dismutase in cells [20], thereby increasing native antioxidant capabilities and decreasing the effects of oxidative stress [4]. The average percent missing outer hair cells in the Protandim Diet group was 55.7 ± 2.43%, and the average percent missing IHCs was 0.48 ± 0.63% per cochlea. Cochleograms for the 499 *prestin* KI mice fed the Protandim diet and the control diet are shown in Fig 3. The overall average percent missing OHCs was not statistically different from the control group (p = 0.33, late generation 499 *prestin* KIs on the Protandim control diet had 57.7 ± 4.4% missing OHCs). However, there does appear to be a trend near the middle of the cochlea the Protandim diet to ameliorate OHC death. This trend reached statistical significance (Student’s t-test) at distances located 30, 37, and 44% from the apex.

### 499 prestin KIs on the FVB genetic background show reduced OHC death

**Fig 4:**
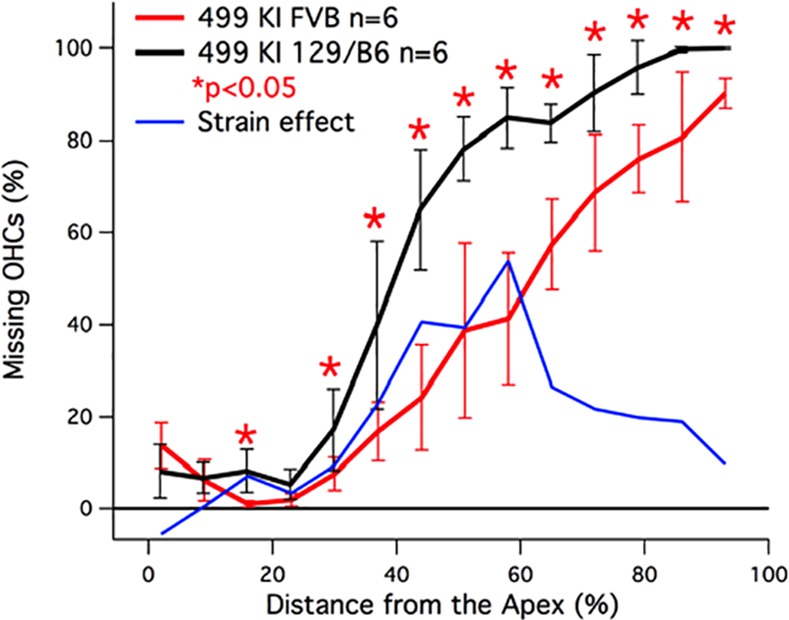
OHC death in 499 *prestin* KIs on two different backgrounds. This graph displays the relationship between the 499 *prestin* KI mice backcrossed onto a FVB strain for 8 generations (red), compared to 499 *prestin* KI mice on the original 129S6/C57BL6 background (black). The backcross was able to significantly attenuate OHC death for 16, 30, 37, 44, 51, 58, 65, 72, 79, 86, 93% sections, as demarcated by the asterisks. The difference between these two plots is appended (blue) to estimate the effect of strain background on OHC survival.

Because genetic background can impact hearing and hair-cell survival, 499 *prestin* KI mice were backcrossed onto the FVB strain for 8 generations since this murine strain maintains good high-frequency hearing with increasing age [36]. The average percent missing outer hair cells in the FVB/499 *prestin* KI group at ~P42 was 35.32 ± 6.05% with no loss of inner hair cells. Cochleograms for 499 *prestin* KIs on the FVB background and their controls on the 129S6/C57BL6 background are shown in Fig 4. The 499 *prestin* KI mice on the FVB background display significantly reduced outer hair cell death over a large region of the cochlea. Although not shown here, the cochleograms obtained at P18 for 499 prestin KI mice on the FVB background were missing only 4% OHCs (n = 4) localized in the basal 20% of the cochlea. This result is better than that for 499 *prestin* KIs on the 129S6/C57BL6 background where 20.9% of the OHCs were missing at ~P18. Also included in Fig 4 is a function (blue) representing the difference between the cochleograms obtained for the two strain backgrounds to estimate the “strain effect”. Since most of the improvement appears in the middle of the cochlea, OHCs in this region may delay their entry into the cell-death pathway.

**Fig 5:**
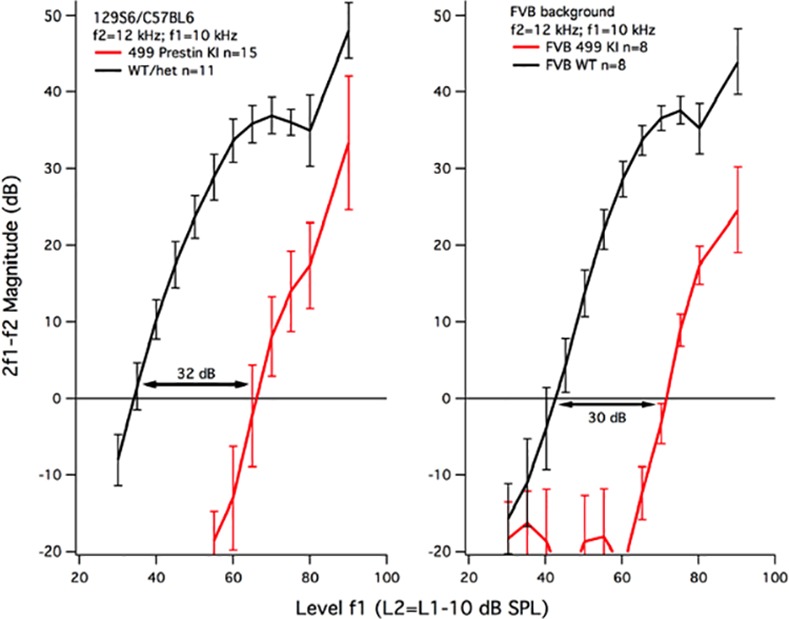
Input-output functions for the DPOAE at 2f1-f2 when f2 = 12 kHz. Data are provided for 499 *prestin* KI mice on the original 126S6/C57BL6 (left) and the FVB backgrounds (right).

Because of the significant improvement in OHC survival seen in the 499 *prestin* KI mice on the FVB background, it was prudent to learn if increased numbers of OHCs correlated with improved hearing. Consequently, we measured otoacoustic emissions, an indicator of OHC function. Input-output functions for DPOAEs at 2f1-f2 are plotted in Fig 5 for f2 = 12 kHz. Independent of strain background, i.e., 129S6/C57BL6 versus FVB, the functions are shifted to the right. When threshold is designated as the level of f1 (L1) required to produce a distortion product of 0 dB, both mouse models suffer threshold shift. For the original 499 *prestin* KI, the WT controls have an average threshold of 34.9 ± 1.8 dB, while the KIs are 67 ± 3.2 dB, giving a threshold shift of 32.1 dB. For the KIs on the FVB strain, the WT control threshold is 41.7 ± 2.7 dB and the FVB/499 KI threshold is 71.3 ± 1 dB with a threshold shift of 29.6 dB. Although the threshold shift is slightly smaller for the FVB/499 *prestin* KIs, it should be noted that the FVB WT controls are about 6 dB less sensitive than the 126S6/C57BL6 controls at this f2 frequency. This difference may relate to the fact that basilar membrane length is on average 5.39 ± 0.13 mm in the FVB strain (n = 6) versus 6.02 ± 0.07 mm for the 129/C57BL6 strain (n = 8). The important point, however, is that the DPOAEs were collected in young mice: the 499 *prestin* KIs on the FVB background were on average 22.5 ± 1.6 days of age, while the KIs on the 129S6/C57BL6 background were 19.3 ± 1.3 days of age. Both groups show a similar reduction in DPOAE magnitude when the average percent missing OHCs is ~4% on the FVB background and ~21% on the 129S6/C57BL6 background.

### Reduced OHC death in compound heterozygous 499 prestin KI/prestin KO mice

**Fig 6:**
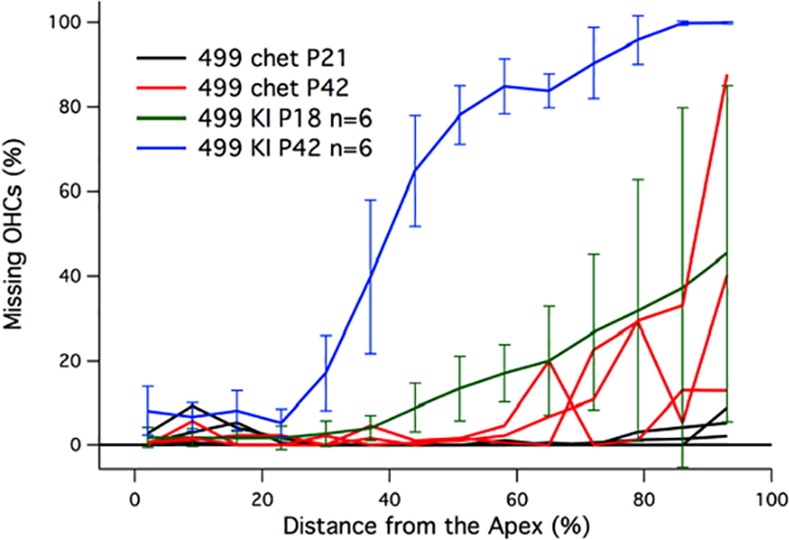
OHC loss observed at various ages. Average cochleograms are provided for the 499 *prestin* KI mice on the original 129S6/C57B6 background at P42 and P18. Also appended are individual plots for three 499 *prestin*/*prestin* KO chets at P21 and P42

The final intervention involved the mating of 499 *prestin* KI mice with *prestin* KOs. In these chets, one chromosome expresses mutated 499 *prestin*, while the second chromosome expresses an allele that does not produce any prestin protein, i.e., it is a functional null. If 499 prestin protein expression is upregulated as it is for WT prestin in *prestin* heterozygotes [37], then the OHCs in this mouse should be near-normal in length and OHC death would be expected to conform to that in the original 499 *prestin* KI. However, if 499 prestin protein in the compound heterozygotes (499 *prestin* KI/*prestin* KO chets) is reduced compared to WT [38], then OHC survival might increase although the cells would be expected to be shorter than normal. In other words, this approach examines the possibility that OHC death is associated with the quantity of 499 prestin protein expressed by the cell. In Fig 6, average cochleograms for 499 *prestin* KIs (129S6/C57BL6) at P42 (blue) and P18 (green) are reproduced from Fig 1. Although OHC preservation is greater in younger mice, large numbers of OHCs are still missing at the base of the cochlea for KIs at P18 and the variability is high. Data for three individual chets at P42 (red) show greater OHC survival when compared to 499 *prestin* KIs at either age. In three chets at P21 (black), very few OHCs are missing. In fact, at P18-21, 499 *prestin* KIs are missing 20.9 ± 11.8% OHCs, while the chets at P21 are missing only 1.47 ± 0.71% OHCs per cochlea. To summarize: a reduction in 499 prestin protein yields a remarkable improvement in OHC survival at both P21 and P42 in the chets.

**Fig 7:**
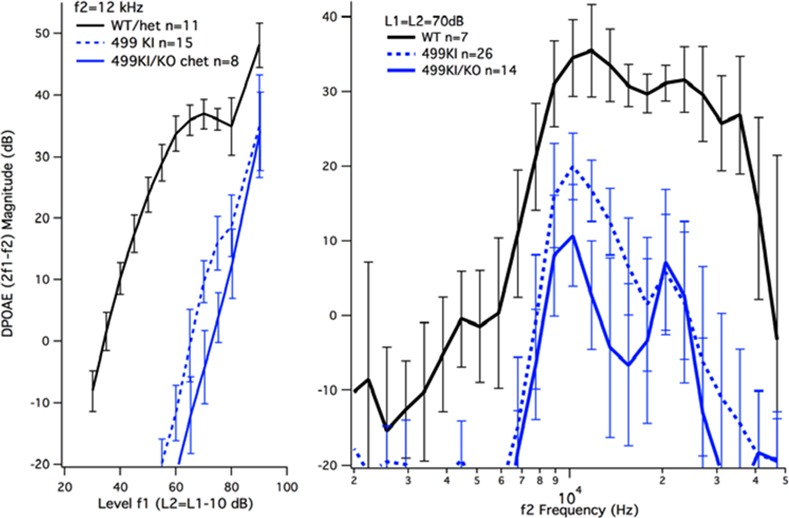
DPOAEs from WT, 499 *prestin* KI (129/B6) and 499 *prestin* KI/*prestin* KO chets. Input-output functions for f2 = 12 kHz (left) and DPgrams for L1 = L2 = 70 dB (right) are provided for the original 499 *prestin* KIs and the chets.

Again, we measured DPOAEs in chets to determine if they had better sensitivity than the 499 *prestin* KIs. As shown in panel A of Fig 7, these mice have reduced DPOAEs re: WT controls (black) and are even slightly worse than the 499 *prestin* KIs (blue dashed lines) for the input-output functions obtained for f2 = 12 kHz. The panel on the right shows magnitude of the DPOAE at 2f1-f2 for L1 = L2 = 70dB plotted as a function of the f2 frequency (f2/f1 = 1.2). Again DPOAE magnitudes for chets are reduced re: WT similar to responses collected from 499 *prestin* KI mice. In spite of the issues relating to cell death in the original 499 *prestin* KIs, the data reported here affirm that the abnormal phenotype is due to the expression of dysfunctional 499 prestin.

### OHC lengths in prestin mouse models

**Fig 8:**
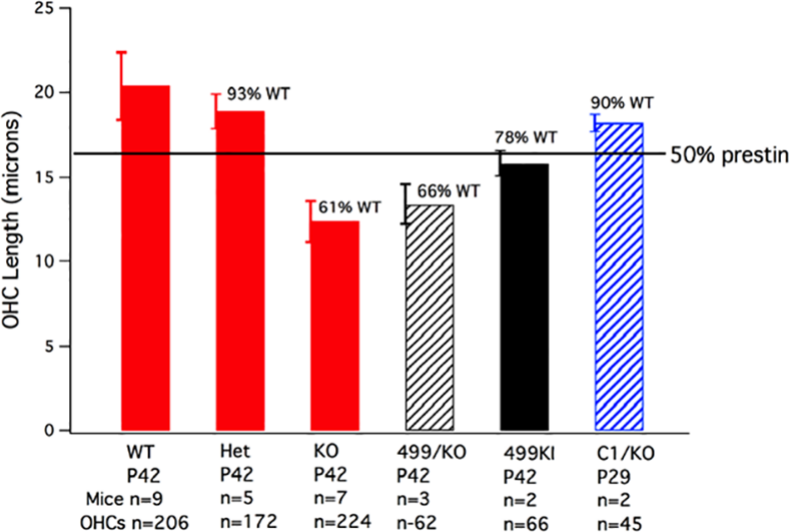
OHC lengths in various *prestin* mouse mutants. Bar graph showing OHC lengths for the original *prestin* KO mouse model [7]. 499 *prestin* KI/*prestin* KO chets are plotted as a black hatched bar, the 499 *prestin* KIs on the 129S6/C57BL6 background as the solid black bar and the C1 *prestin* KI/*prestin* KO chets as the hatched blue bar. The horizontal line indicates predicted length of OHCs if prestin expression were to be reduced by 50% (see the main text). In all cases, OHC lengths in these various mutants were statistically different from WT, with all p values being less than p = 0.01 as determined using the Student’s t-test.

Since there was a large improvement in OHC survival when only one copy of 499 *prestin* was expressed, we measured OHC lengths in chets to see if they were shorter than in WT controls, but longer than in KOs, which might be expected if ~50% 499 prestin protein were being generated. Data provided in Fig 8 were obtained for OHCs located 2–2.1 mm from the helicotrema (the ~13 kHz place) [24] and include our results from the original *prestin* KOs along with companion data from WT and heterozygous mice (red bars). These results showed upregulation of WT prestin protein in *prestin* hets since the cells were 93% of WT in length. However, the OHCs in *prestin* KOs were much shorter [7], implying that prestin is an important structural protein. This reduction in length was expected since prestin protein occupies much of the OHC’s lateral membrane in normal animals and since OHC length decreases in a prestin-dependent manner [39]. We reasoned that if OHCs with no prestin protein were ~12 micrometers (μm) in length and WT were ~20 μm, a heterozygote producing 50% protein would be ~16 μm long as shown by the horizontal line. Also included in the figure are the average OHC lengths in the 499 *prestin* KI/*prestin* KO chet (black hatched bar). These OHCs are shorter than a *prestin* heterozygote with one copy of WT *prestin*.

Because OHCs in 499 *prestin* KI/*prestin* KO chets were short, we examined OHC lengths in 499 *prestin* KIs on the original 129S6/C57BL6J background. The solid black bar indicates that OHCs in these mice are shorter than expected, i.e., 78% of WT. However, when the measurements were taken at P21 instead of P42, the 499 *prestin* KI OHCs were 89% of WT at the 2.1 mm location. At 0.5 mm from the helicotrema (~6 kHz), 499 *prestin* KI OHCs at P21 were 93% of WT. This increase in length at younger ages and at more apical locations may reflect the basal to apical progression of OHC death. In other words, OHCs in younger animals at more apical locations may be in better condition and, therefore, closer to WT in length. 499 *prestin* KI/*prestin* KO chets show a similar progression although much less dramatic: at P21 chets are 68% of WT in length at 2.1 mm from the helicotrema and 82% of WT at 0.5 mm from the helicotrema.

OHC lengths were also measured in C1 *prestin* KI/*prestin* KO chets. In C1 *prestin* KI mice where no OHC death is observed, NLC is normal except for the fact that prestin’s voltage dependence is shifted in the hyperpolarizing direction [21,22]. Sensitivity and frequency selectivity are also WT-like as indicated by recordings of the compound action potentials. In these C1/KO chets, the OHCs were 90% of WT at P29 (blue hatched bar), indicating enhanced C1 mutated prestin protein expression since the cells were longer than ~16 microns. The fact that OHC length is near normal in C1 *prestin* KI/*prestin* KO chets contrasts with data obtained from the 499 *prestin* KI/*prestin* KO chets where the OHCs were only 66% of WT in length. This observation implies that 499 prestin may be toxic to the cell. If true, then the death process in 499 KIs may differ from that in *prestin* KO mice.

## Discussion

499 *prestin* KI mice show accelerated, progressive OHC death when compared to both *prestin* KO and wildtype mouse models expressing age-related hearing loss genes (129/C57BL6). This observation was surprising and suggests that perhaps the mechanisms of OHC death may not be the same in mice lacking prestin versus those expressing mutant 499 *prestin*. In this study, we attempted to attenuate the effects of dysfunctional prestin in the 499 *prestin* KI mouse by administering a variety of treatments aimed to ameliorate OHC loss in order to make the 499 *prestin* KI mouse model more suitable for studying cochlear amplification. Our results indicate that 499 *prestin* KI mice, which were also homozygous for the *Bak* gene, failed to show any improvement in OHC death. Potential explanations may relate to the molecular mechanisms underlying AHL, which are largely unknown. Although it was hypothesized that mice lacking the *Bak* gene would have a reduced tendency to enter the apoptotic cell death pathway, Bak-dependent mitochondrial apoptosis is probably not solely responsible for progressive OHC death. In addition, experiments in zebrafish showed that differential regulation of *Bax* and *Bcl2* revealed a complex interplay of pro-death/pro-survival proteins in drug induced hair cell loss [34], attesting to the intricate relationships between various genetic factors. This possibility is also emphasized in recent reviews [14,30] where it is stated that damage from any given event probably relates to a balance between various cell death mechanisms including both intrinsic (for example, mitochondrial) and extrinsic (death receptor mediated) apoptosis, necrosis and necroptosis. Although there was some improvement using the Protandim diet, it was small and the average number of missing OHCs per cochlea in the treated group was not significantly reduced relative to controls. Perhaps the diet did not reduce oxidative stress enough to protect OHCs or a time interval longer than 6 weeks is required. Taken together, these outcomes are consistent with other failed efforts using anti-oxidants (d-methionine, vitamin supplementation) and compounds known to interfere with apoptosis (minocycline) to improve OHC survival in 499 *prestin* KI mice.

In contrast, 499 *prestin* KIs on the FVB background showed a significant reduction in OHC death (Student’s t-test) at ~6 weeks of age. This outcome suggests that the expression of 499 mutated *prestin* coupled with the known AHL in these mouse strains, may predispose OHCs to enter the cell-death pathway(s) earlier than normal. It is unlikely, however, that the attenuation of hair cell death is solely related to a reduction in oxidative damage since anti-oxidant supplementation failed to induce large improvements in OHC survival. In other words, an increase in the production of antioxidant enzymes is probably insufficient to explain the dramatic improvement in OHC survival for the 499 *prestin* KIs on the FVB background.

Creation of chets where one allele is a *prestin* null and the other produces 499 mutated *prestin* also improved OHC survival. However, in this case the OHCs were shorter, which contrasts with results obtained from the C1 *prestin* KI/*prestin* KO chets where OHCs were 90% of WT in length, consistent with auto regulation of C1 prestin protein expression. If 499 prestin is deleterious to cell survival, then reduced expression levels may underlie the improvement in OHC survival in 499 *prestin* KI/*prestin* KO chets.

The improvement in OHC numbers observed in 499 *prestin* KI mice backcrossed to FVB and in 499 *prestin* KI/*prestin* KO chets did not increase sensitivity. The DPOAE thresholds in 499 *prestin* mouse models on both the 129S6/C57BL6 and FVB backgrounds were shifted ~30dB, as were those in the chets with minimal OHC loss. These results attest to the fact that prestin is required for cochlear amplification [2,40,41]. The emission data were collected on mice around weaning to maximize the numbers of surviving OHCs. Even when only a small percentage of the OHCs were missing in young 499 *prestin* KIs on the FVB background, the decrease in DPOAE magnitude was similar to that recorded in the original mouse strain. This result is similar to that observed when the *prestin* KO mouse was backcrossed to the CBA/CaJ mouse strain. In this case [42], the improvement in OHC survival at P42 did not change hearing phenotype, i.e., *prestin* KO mice on both backgrounds (129S7/C57BL6 or CBA/CaJ) showed a similar loss of sensitivity and the compound action potential (CAP) tuning curves lacked sharply-tuned tip segments. The observation that DPOAEs did not change dramatically in 499 *prestin* KIs when OHC survival increased is also consistent with recent reports in the literature. For example, optical coherence tomography [41] and laser vibrometry [40] were used to make mechanical measurements *in vivo*. Both studies showed no cochlear amplification and broad tuning in mice expressing 499 mutated *prestin*. In spite of these reports, however, it is possible that a small gain is provided by the hair bundle amplifier but that this contribution is difficult to identify.

Since OHC loss is the most common form of cellular damage in the organ of Corti, the treatment conditions examined in this report provide insights into the precautions that can be taken to attenuate OHC death within the mammalian cochlea. Our results indicate that premature OHC loss in 499 *prestin* KI mice appears to be induced intrinsically in a *Bak*-independent manner. In addition, the severity of OHC loss depends on genetic background and only slightly improves using an anti-oxidant diet. These results imply that genetics, age-related hearing loss, and oxidative stress interact in complex ways to influence OHC survival. Furthermore, it is provocative that a simple modification involving only 2 amino acids in the *prestin* gene can produce toxic effects, while the presence of normal, WT prestin is important for OHC survival.
